# Allelic variations of the *Wx* locus in cultivated rice and their use in the development of hybrid rice in China

**DOI:** 10.1371/journal.pone.0232279

**Published:** 2020-05-05

**Authors:** Ye Shao, Yan Peng, Bigang Mao, Qiming Lv, Dingyang Yuan, Xionglun Liu, Bingran Zhao

**Affiliations:** 1 College of Agronomy, Hunan Agricultural University, Changsha, China; 2 State Key Laboratory of Hybrid Rice, Hunan Hybrid Rice Research Centre, Changsha, China; Louisiana State University College of Agriculture, UNITED STATES

## Abstract

To make better use of global germplasm resources for improving the eating quality of hybrid rice, using the resequencing data from the 3,000 rice genomes project (3K RGP), the allelic variations of the rice *Wx* locus were analysed. With the exception of five rare alleles discovered for the first time in our study, most of these alleles were known alleles of *Wx*. Furthermore, a set of Kompetitive allele-specific PCR (KASP) markers based on these *Wx* alleles have been developed, and thirty-six main parents of hybrid rice from 1976 to 2018 were selected for *Wx* genotyping. The results showed that only three *Wx* alleles existed in the main parents of hybrids, and the allelic combination of the hybrids changed from *Wx*^*a*^/*Wx*^*b*^ and *Wx*^*lv*^/*Wx*^*b*^ to *Wx*^*b*^/*Wx*^*b*^ with the development of hybrid rice. *Wx*^*b*^ is widely used in the male parents of hybrid rice. *Wx*^*a*^ and *Wx*^*lv*^ were used in the female parents of early hybrid rice, and they were gradually replaced by *Wx*^*b*^. In the future, more favourable *Wx* alleles from cultivated rice should be identified, introduced, and effectively used to improve hybrid rice quality.

## Introduction

Rice is one of the most important crops with the great success in heterosis applications. For a long time, considerable attention has been given to improve yield in hybrid rice. But research on rice quality started fairly late in China, especially in southern areas. With the improvement in the standard of living, consumers are more concerned about rice quality. Over the past 15 years, the quality traits of *indica* hybrid rice have improved to a certain extent. However, further improvements are still required to make hybrid rice comparable to high-quality conventional rice, especially in terms of eating quality [1,2]. As many widely used hybrid parents are derived from the same basic materials, the genetic polymorphisms in hybrid rice may be relatively low [3]. Thus, an improved understanding the diversity of genes associated with eating quality in cultivated rice and hybrid rice is needed for improving the quality of hybrids.

Rice quality is a complex characteristic, with amylose content (AC) serving as a key determinant of eating and cooking quality [4,5]. The *Wx* gene, which encodes a granule-bound starch synthase (OsGBSS1), is a critical gene controlling AC. The transcription and OsGBSS1 protein activity of this gene are positively correlated with AC [6]. To date, at least eight *Wx* alleles have been identified in rice—*Wx*^*a*^, *Wx*^*b*^, *Wx*^*in*^, *Wx*^*op*^, *Wx*^*mq*^, *Wx*^*mw*^, *Wx*^*lv*^, and *wx*. The AC of these alleles decreases in the following order: *Wx*^*lv*^ (>25%), *Wx*^*a*^ (20–25%), *Wx*^*in*^ (18–22%), *Wx*^*b*^ (15–18%), *Wx*^*mw*^ (10–14%), *Wx*^*mq*^ (8–12%), *Wx*^*op*^ (5–10%), and *wx* (AC<2%) [7–13]. However, whether there exist some new functional alleles in cultivated rice resources worldwide has not been elucidated to date. Moreover, we hope to develop a set of molecular markers for high-throughput genotyping in the *Wx* locus, but whether the known alleles can cover most of the *Wx* allelic variations and explain variation in AC has not been determined.

In 2015, 3,000 germplasm accessions from 89 different countries/regions were selected for genome-wide resequencing, and a comprehensive SNP and InDel sub-database was established for the Rice Functional Genomics-based Breeding (RFGB) Database [14]. Of these accessions, 2,466 were retrieved from a core collection of over 101,000 rice accessions in the International Rice Genebank Collection (IRGC), while 534 accessions were selected from a core collection of 61,470 rice accessions preserved in the China National Crop Genebank (CNCGB) [15]. Thus, the 3,000 germplasm accessions represent a panel with abundant genetic diversity worldwide. Using the currently available genome information from the 3,000 rice genomes project (3K RGP) sequencing data, allelic variations at the *Wx* locus can be comprehensively analysed, and new allelic variants responsible for different AC classes can be discovered to improve the quality of hybrid rice.

A prior study revealed a significant correlation between cytosine and thymidine (CT) repeat and AC, and microsatellite markers have been developed [16]. However, some of the AC variations cannot be accurately explained by these markers [17]. With a series of *Wx* alleles identified in rice [18], some SNP genotyping methods have been used for polymorphism analysis of the *Wx* locus. Kompetitive allele-specific PCR (KASP) is a high-throughput method that can be employed for SNP and InDel genotyping at specific sites. Different genotypes at a single site can be detected by two-colour fluorescence based on terminal fluorescence reading [19]. Therefore, owing to advantages such as high efficiency, accuracy, and throughput, the KASP marker may help accelerate rice quality breeding.

In this study, to make better use of the *Wx* allele resources and breed hybrid rice with improved eating quality, we investigated the diversity of *Wx* alleles in cultivated rice and their development in hybrid rice. Using the 3K RGP resequencing data, we identified not only eight known alleles that existed widely across the globe but also five allelic variations that had not been previously reported. Furthermore, a set of KASP markers based on these *Wx* alleles was developed, and thirty-six main parents of hybrid rice were selected for *Wx* genotyping. We found only three *Wx* alleles existed in these main parents, and the allelic combination of hybrid rice changed from *Wx*^*a*^/*Wx*^*b*^ and *Wx*^*lv*^/*Wx*^*b*^ to *Wx*^*b*^/*Wx*^*b*^ as the quality improved. Because the allelic variation of the *Wx* locus in hybrid rice is relatively low, more favourable *Wx* alleles from cultivated rice should be introduced for further quality breeding of hybrid rice.

## Materials and methods

### Plant materials

By referring to previously described data [20], the Hybrid Rice Variety Resources Database (http://www.hybridrice.com.cn/), and the China Rice Data Centre (http://www.ricedata.cn/), thirty-six main parents of hybrid rice from 1976 to 2018 were selected for *Wx* genotyping. All the combinations of these parents are the hybrid rice with the largest planting area over the years. The hybrid parents included eighteen female parents of Erjiunan 1A, Zhenshan 97A, V20A, Gang 46A, Longtefu A, Bo A, II-32A, Xieqingzao A, Jin 23A, Zhong 9A, Tianfeng A, Annong-S1, Peiai 64S, Guangzhan 64-4S, Y58S, Zhu 1S, C815S, Longke 638S and eighteen male parents of IR24, IR26, Minghui 63, Ce 64–7, Duoxi 1, Fuhui 838, Xianhui 207, Shuhui 527, CDR22, Miyang 46, R402, Minghui 86, Mianhui 725, Gui 99, Guanghui 3550, Yangdao 6, Bing 4114, Huazhan. The control group included Zhenshan 97A (*Wx*^*a*^), Nipponbare (*Wx*^*b*^), Kasalath (*Wx*^*lv*^), Basmati (*Wx*^*in*^), Haopi (*Wx*^*op*^), and Nanjing 46 (*Wx*^*mq*^). All hybrid parental lines and control materials for genotyping were stored in our laboratory. Four germplasm resources—IRIS_313–9445, IRIS_313–10892, IRIS_313–10866, and IRIS_313–8956—were obtained from the Crops Research Institute, Chinese Academy of Agricultural Sciences. All varieties were cultivated under normal growing conditions in the experimental field of the Hunan Hybrid Rice Research Centre in Changsha.

### Resequencing data of the *Wx* alleles

The region for sequence analysis is located in a region (1765622–1770574) on chromosome 6 according to the Nipponbare genome (IRGSP V.1). Data for the SNPs and InDels at the *Wx* locus in 3,000 rice accessions were downloaded from the Rice SNP-Seek Database (http://oryzasnp.org/iric-portal/index.zul).

### Sequence analysis

Sequences were aligned using CLUSTAL X version 2.0 and adjusted manually with Microsoft Office Excel 2010 [21]. Haplotype diversity was calculated using DNASP v5.0 [22], and the haplotype network was constructed using PopART 1.7 [23]. A geographical distribution map of the *Wx* alleles was generated using rworldmap V1.36 [24].

### Crystal structure analysis

The crystal structure of the rice OsGBSSI catalytic domain in a complex with ADP was downloaded from the Protein Data Bank (http://www.rcsb.org/pdb/). The PyMOL Molecular Graphics System (Schrödinger LLC) was used to display the structural features of the OsGBSSI protein (PDB: 3VUF) with a focus on the novel mutations identified.

### Detection of the five allelic variations by Sanger sequencing

Genomic DNA was extracted from fresh leaves of the IRIS_313–9445, IRIS_313–10892, IRIS_313–10866, and IRIS_313–8956 varieties using a modified CTAB method. Briefly, a 266-bp sequence containing the mutation site of Ex2+160 was amplified using the primer pair 5′-ATGTCGGCTCTCACCACG-3′ and 5′-CCGACGAACACGACGTTCATG-3′; a 297-bp sequence containing the mutation site of Ex4+73 was amplified using the primer pair 5′-GATACCAGCGTTGTGGCTGAG-3′ and 5′-CAGTCCAACTGCTAAATGCACTG-3′; a 194-bp sequence containing the mutation site of Ex14+2 was amplified using the primer pair 5′-GAGTGACAAATTTCAGGCAATCGAG-3′ and 5′-CCAGAAGAACGATCTGGACGTC-3′; and a 174-bp sequence containing the mutation sites of Ex14+2 and Ex14+28 was amplified using the primer pair 5′-CAGAGATTCACCTGTCTGATGCTG-3′ and 5′-TCAAGGAGCAGCCACGTTCTC-3′. PCR amplification was carried out as follows: initial DNA denaturation at 95 °C for 4 min; 30 cycles of denaturation at 95 °C for 30 s; annealing at 58 °C for 30 s; extension at 72 °C for 30 s; and final extension at 72 °C for 5 min. After gel purification, the PCR products were sequenced by TsingKe Biology Technology, Bei Jing, China.

### KASP genotyping

The allele-specific primers were designed to carry the standard FAM (5′GAAGGTGACCAAGTTCATGCT3′) and HEX (5′ GAAGGTCGGAGTCAACGGATT 3′) tails and the targeted SNP at the 3′ end. Assays were carried out in 384-well formats and 10-μl reactions (20–30 ng/μl DNA, 5 μl of 1× KASP master mixture, 0.14 μl of KASP assay mix, and 4.86 μl of water). PCR was conducted using the following protocol: hot start at 94 °C for 15 min, ten touchdown cycles (94 °C for 20 s; initial touchdown at 61 °C and then a decrease by −0.6 °C per cycle for 60 s), and 26 additional cycles of annealing (94 °C for 20 s; 55 °C for 60 s). Finally, the PCR product with fluorescent labelling was scanned using a Roche Light Cycler 480.

### Amylose content measurement

The AC of rice seeds was measured using the iodine colorimetry assay described previously [25].

## Results

### Analysis of *Wx* allelic variations in 3K RGP

Using the coding sequence (CDS) of Nipponbare as a reference, allelic variations in the coding region of the *Wx* gene were analysed according to the 3K RGP sequencing data. After eliminating the alleles with heterozygous sequences in the coding region and the possible deletion caused by insufficient sequencing coverage, a total of 2,752 lines were obtained for variation analysis. Based on the G/T SNP at the splice site of the first intron (In1G/T SNP), two important alleles, namely, *Wx*^*a*^ and *Wx*^*b*^, have been identified previously [26]. Although it does not belong to the coding region, this functional SNP should be taken into account for analyzing variation.

**Fig 1:**
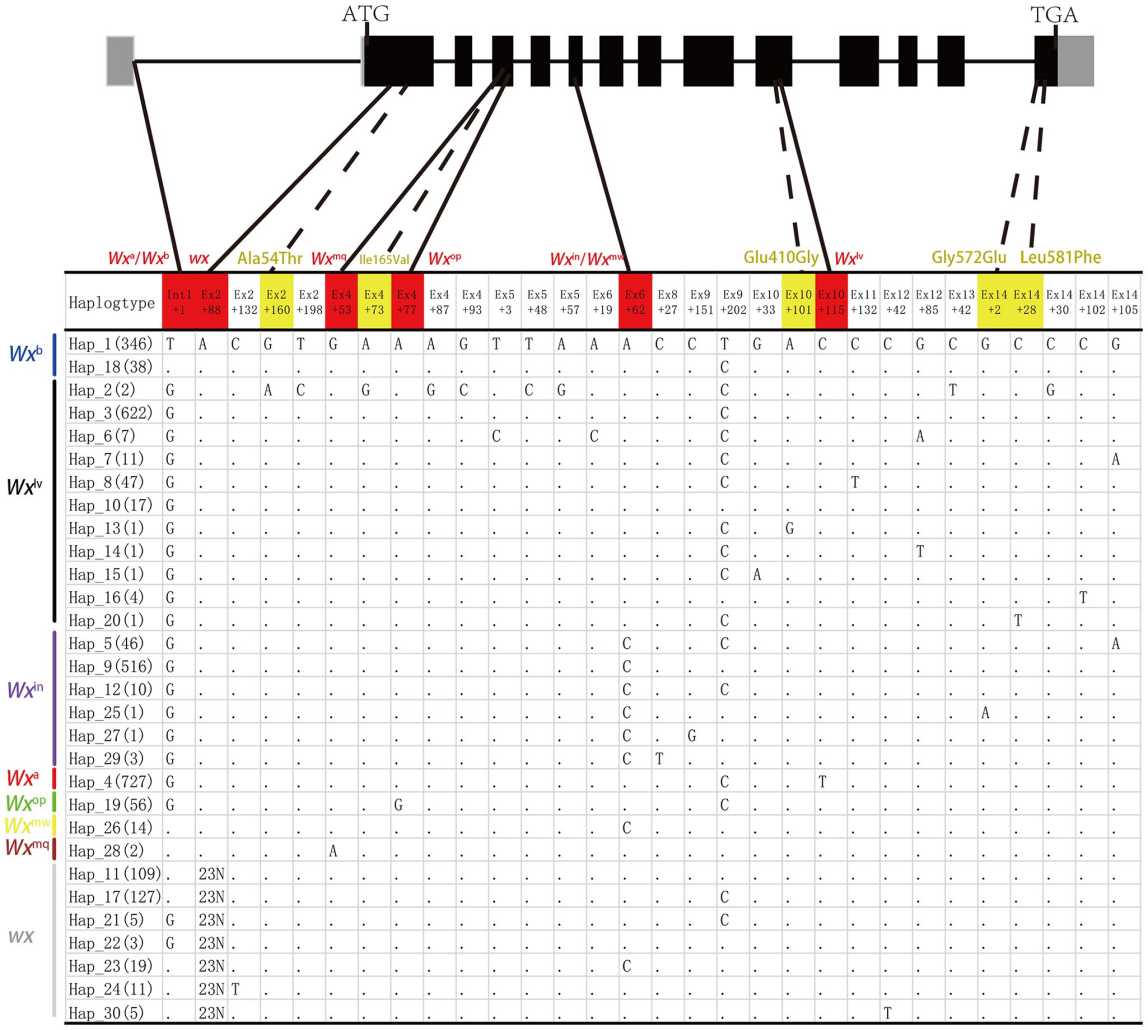
Analysis of the allelic variation among the 2,752 lines. Each vertical line with a unique colour represents the same type of allele. Coloured boxes represent non-synonymous mutations; red box: mutation sites of each known allele, yellow box: mutation sites of each unreported allele. Boxes without colour represent synonymous mutations. Numbers in parentheses represent the total number for each specific haplotype. Hap1 (346) including 345 lines from 3 K RGP and one control material (Nipponbare).

**Fig 2:**
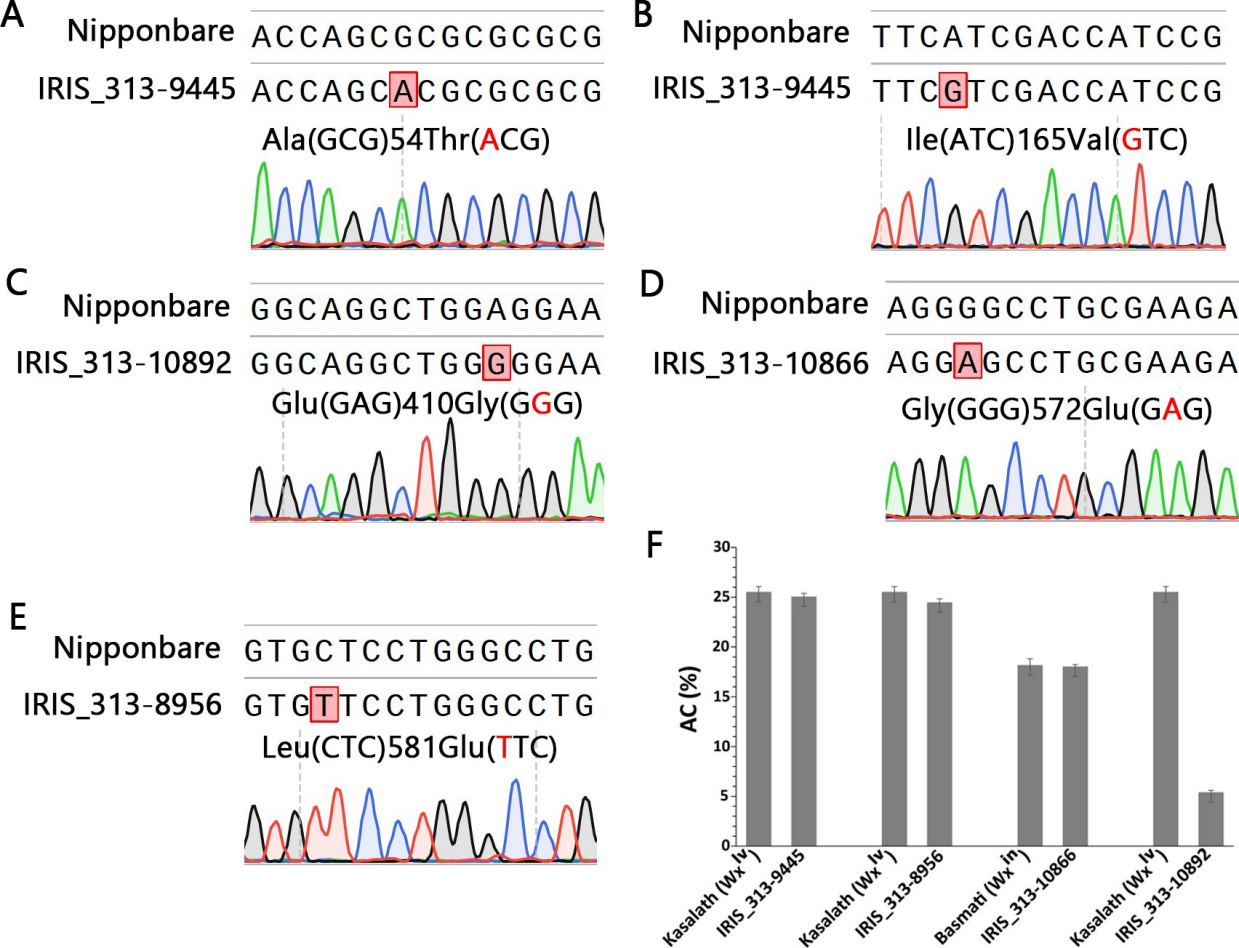
DNA sequence validation and AC measurement. (A-E) Sequencing validation of five non-synonymous mutations. (F) Amylose content in mature seeds of four IRIS lines and WT (*Wx*^*lv*^ and *Wx*^*in*^). Kasalath (*Wx*^*lv*^) and Basmati (*Wx*^*in*^) were used as the control group.

**Fig 3:**
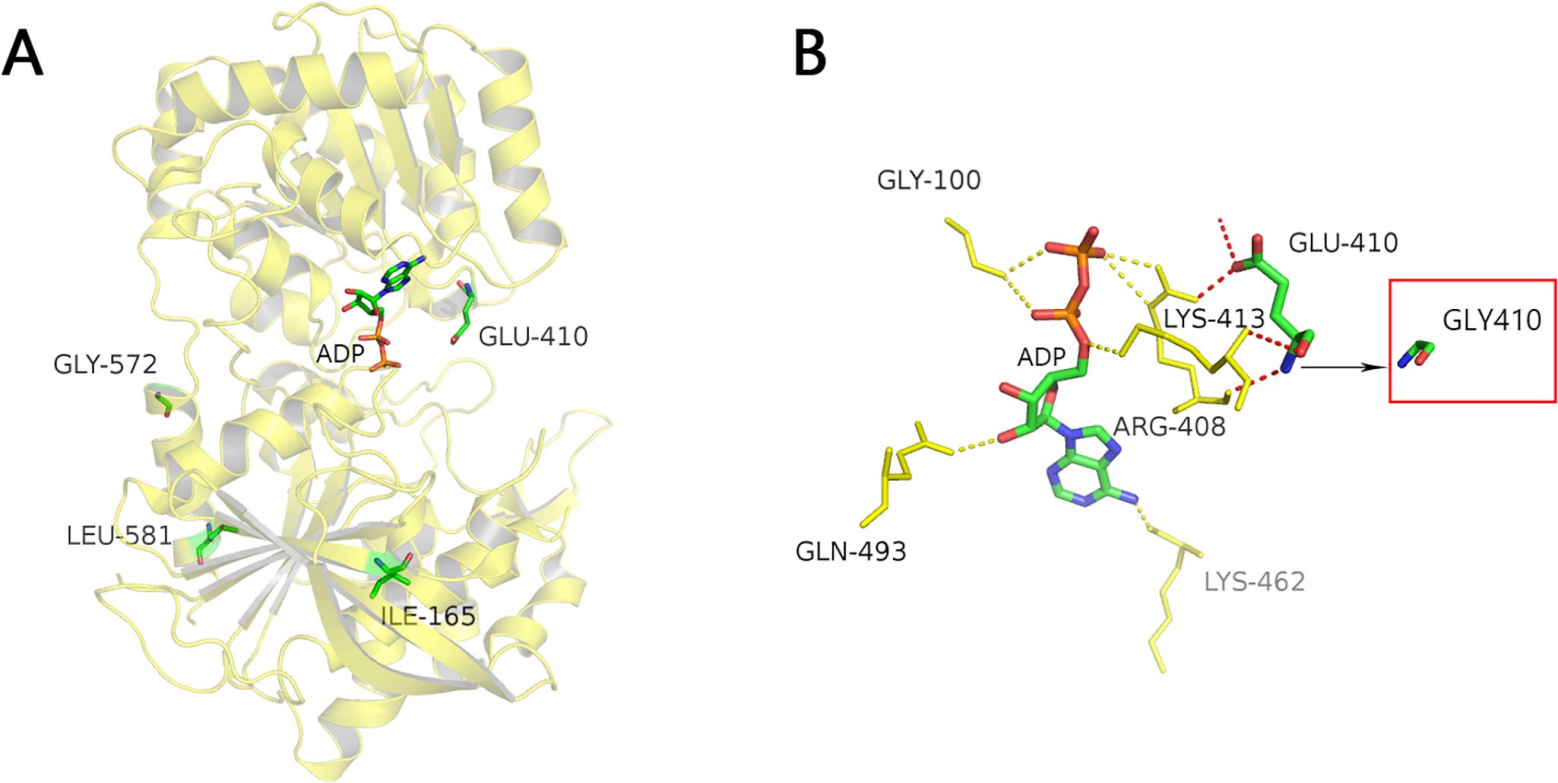
Crystal structure of rice OsGBSS1. (A) Crystal structure of the OsGBSSI catalytic domain in complex with ADP. ADP, Glu410, Gly572, Leu581, and Ile165 are each represented by the highlighted stick models. (B) The enlarged active centre of OsGBSS1. ADP is located in the middle area, the five amino acids linked to ADP by hydrogen bonds are indicated by yellow sticks, and the dotted yellow line represents the hydrogen bonds between them. The dotted red line represents the hydrogen bonds between Glu410 and two important amino acids. Gly410 in the red box represents mutated amino acids.

Based on 1 insertion and 29 nucleotide polymorphic sites (SNPs), a total of 30 haplotypes of *Wx* alleles were identified (Fig 1). Among these haplotypes, Int1+1, Ex2+88, Ex4+53, Ex4+77, Ex6+62, and Ex10+115 formed the eight known alleles, representing 99.9% of the allelic variation in the 2,752 lines. In addition, five non-synonymous mutations—Ex2+160, Ex4+73, Ex10+101, Ex14+2, and Ex14+28—were identified for the first time, and they resulted in amino acid changes from Ala to Thr (54 cases), Ile to Val (165), Glu to Gly (410), Gly to Glu (572) and Leu to Phe (581). Ex2+160 and Ex4+73 coexisted in IRIS_313–9445, Ex10+101, Ex14+2 and Ex14+28 existed in IRIS_313–10892, IRIS_313–10866 and IRIS_313–8956, respectively. Except for these novel mutations, IRIS_313–9445, IRIS_313–8956 and IRIS_313–10892 contained the *Wx*^*lv*^ allele (AC≈25%), and IRIS_313–10866 contained the *Wx*^*in*^ allele (AC≈18%). By determining the AC in the mature seeds of these four IRIS lines, we found that except for IRIS_313–10892 (Ex10+101), the AC of the other three materials remained generally unchanged compared with that of the wild type (Fig 2). By analysing the crystal structure of OsGBSS1, we found that Glu410Gly (Ex10+101) is located in the active centre of the enzyme, next to the ligand ADP of OsGBSS1, while the remaining four mutation sites were located at a distance from the active centre (Fig 3B). In addition, Ala54Thr did not even exist in the truncated body of OsGBSS1.

### Construction of the *Wx* haplotype flowchart

**Fig 4:**
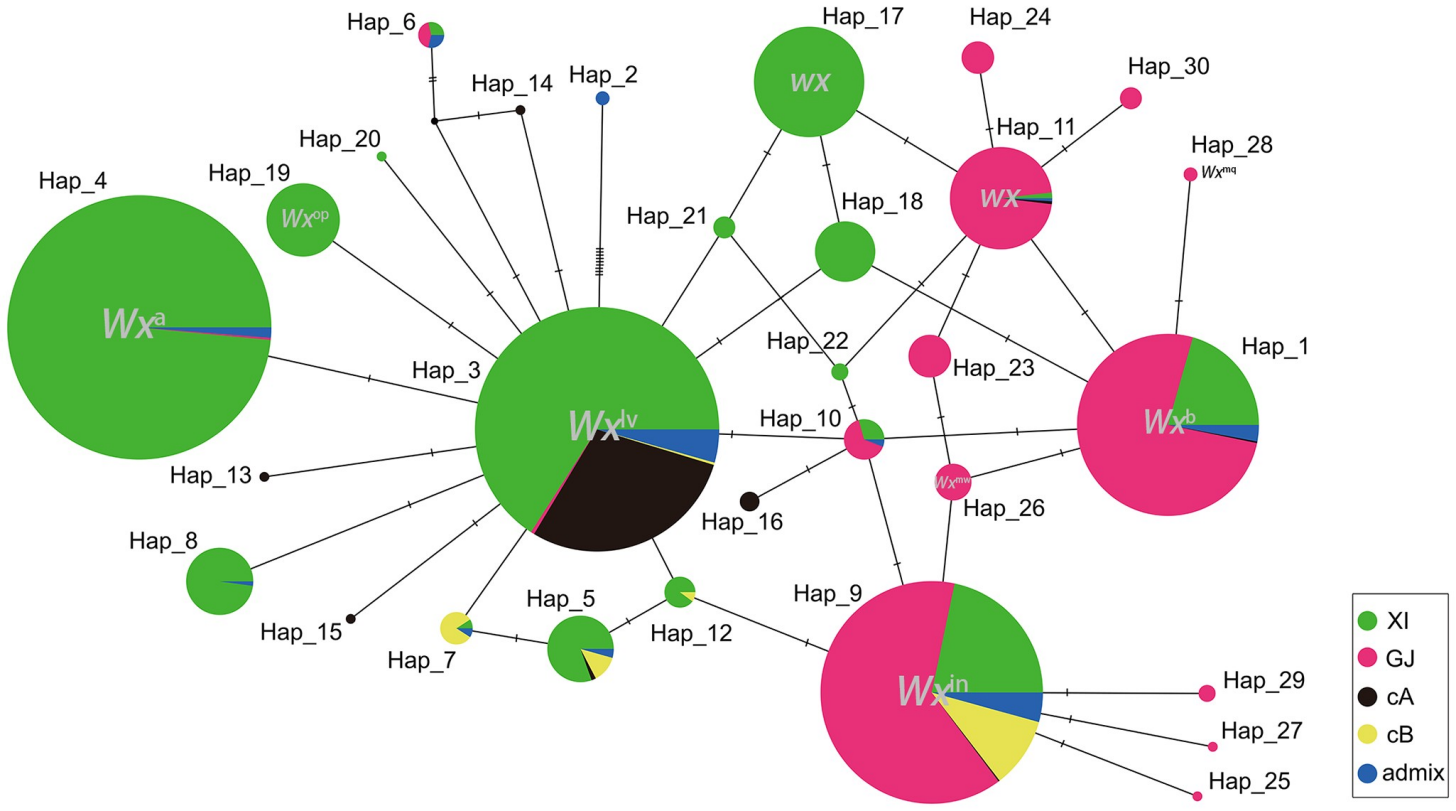
Flowchart of the 30 *Wx* haplotypes. Different colours represent different varietal groups. XI, GJ, cA, cB, and admix represent the *Xian*/Indica, *Geng*/Japonica, circum-Aus, circum-Basmati, and intermediate groups, respectively [27]. Each pie chart indicates a unique haplotype and the proportions of different varietal groups. The size of each pie chart represents the number of lines in each haplotype. Hap1, Hap3, Hap4, and Hap9 represent *Wx*^*b*^, *Wx*^*lv*^, *Wx*^*a*^, and *Wx*^*in*^, respectively. Each short line segment on a solid line represents one SNP between two haplotypes.

To understand the genetic background of each haplotype and the relationships among them, a haplotype flowchart was constructed to describe the mutational steps of 30 haplotypes. Hap1, Hap3, Hap4, and Hap9, which represent *Wx*^*b*^, *Wx*^*lv*^, *Wx*^*a*^, and *Wx*^*in*^, respectively (Fig 4), demonstrated absolute predominance compared to the other haplotypes in cultivated rice. With the exception of *wx*, *indica*-*japonica* background differences existed among the remaining seven alleles. Notably, the *Wx*^*a*^, *Wx*^*lv*^, and *Wx*^*op*^ alleles belonged to the *indica* background, while *Wx*^*b*^, *Wx*^*in*^, *Wx*^*mw*^, and *Wx*^*mq*^ represented the *japonica* background. *Wx*^*lv*^ was recognized as the predominant allele in the cA subgroup, while *Wx*^*in*^ is the predominant allele of cB. These findings indicated that *Wx* alleles have regional preference and a corresponding environmental adaptability.

### Worldwide distribution of different *Wx* alleles

**Fig 5:**
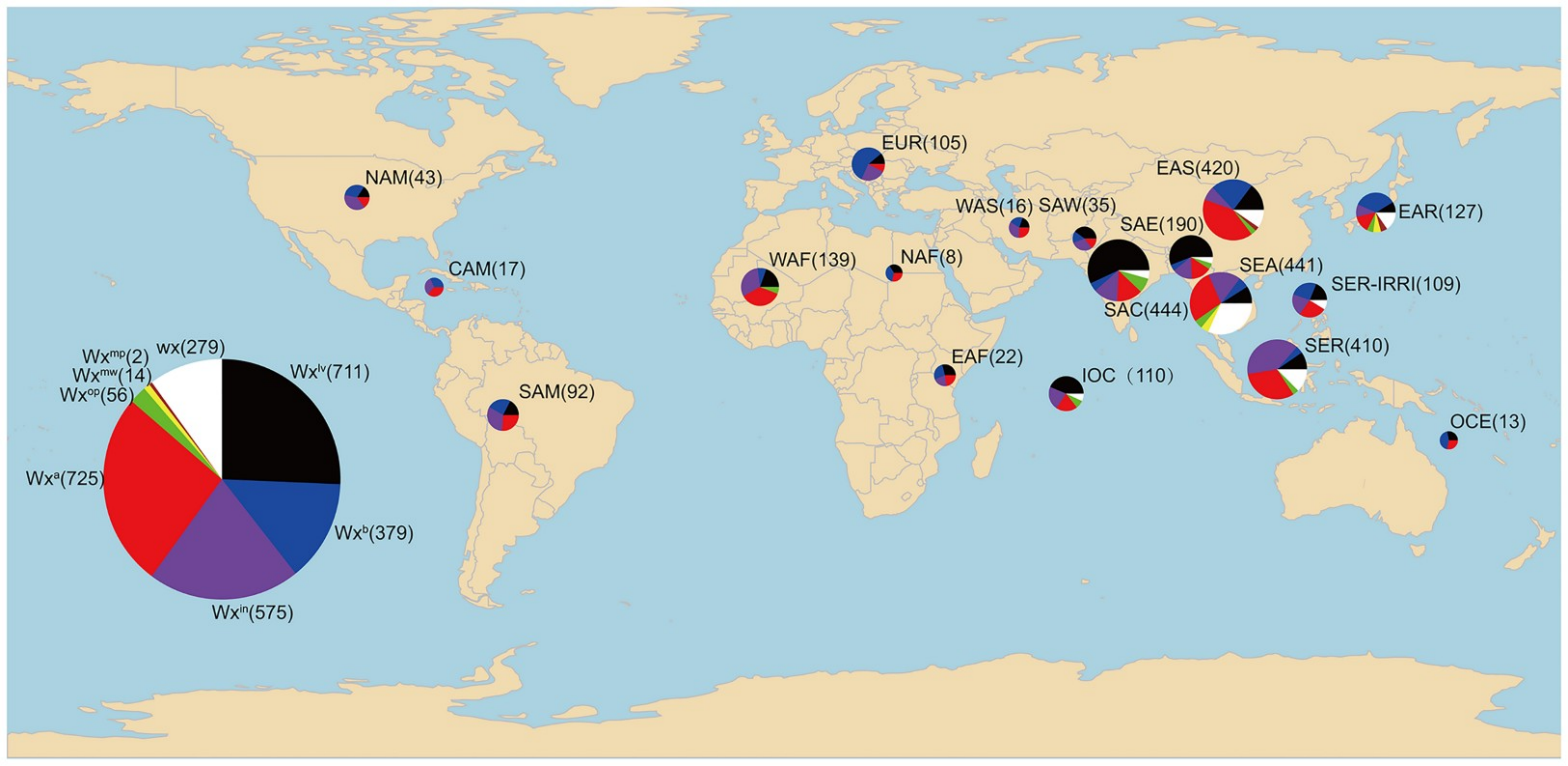
Worldwide distribution of different *Wx* alleles. Each colour represents a unique *Wx* allele. Red: *Wx*^*a*^, black: *Wx*^*lv*^, purple: *Wx*^*in*^, blue: *Wx*^*b*^, white: *wx*, green: *Wx*^*op*^, yellow: *Wx*^*mw*^, and red-brown: *Wx*^*mq*^. Each pie chart indicates the proportions of different *Wx* alleles in a certain area, and the numbers in parentheses indicate the sample size for each pie chart. The pie charts at the bottom left indicate the proportions of different *Wx* alleles in 3K RGP, and the numbers in parentheses indicate the numbers of a certain *Wx* allele.

A global map showing the distributions of the eight known alleles based on the 3k RGP information (Fig 5) indicates that *Wx*^*a*^, *Wx*^*b*^, *Wx*^*in*^, and *Wx*^*lv*^ are four predominant alleles with a wide distribution in most rice-producing regions of the world. However, regional differences existed among the alleles. Compared with other alleles, *Wx*^*b*^ is widely distributed at high latitudes, *Wx*^*in*^ is widely distributed in tropical areas at low latitudes, and *Wx*^*a*^ is located in the mid-latitude region. In addition, *Wx*^*lv*^ was mainly distributed in the South Asia-Central (SAC) and South Asia-East (SAE) areas, and *wx* was commonly distributed in Southeast Asia (SEA) and East Asia (EAS). These findings are consistent with the eating habits of the regions, as high-amylose varieties are popular in Myanmar, Sri Lanka, provinces of Indonesia, and many states of India [28].

### Correlation analysis between the *Wx* alleles and CT repeats

**Fig 6:**
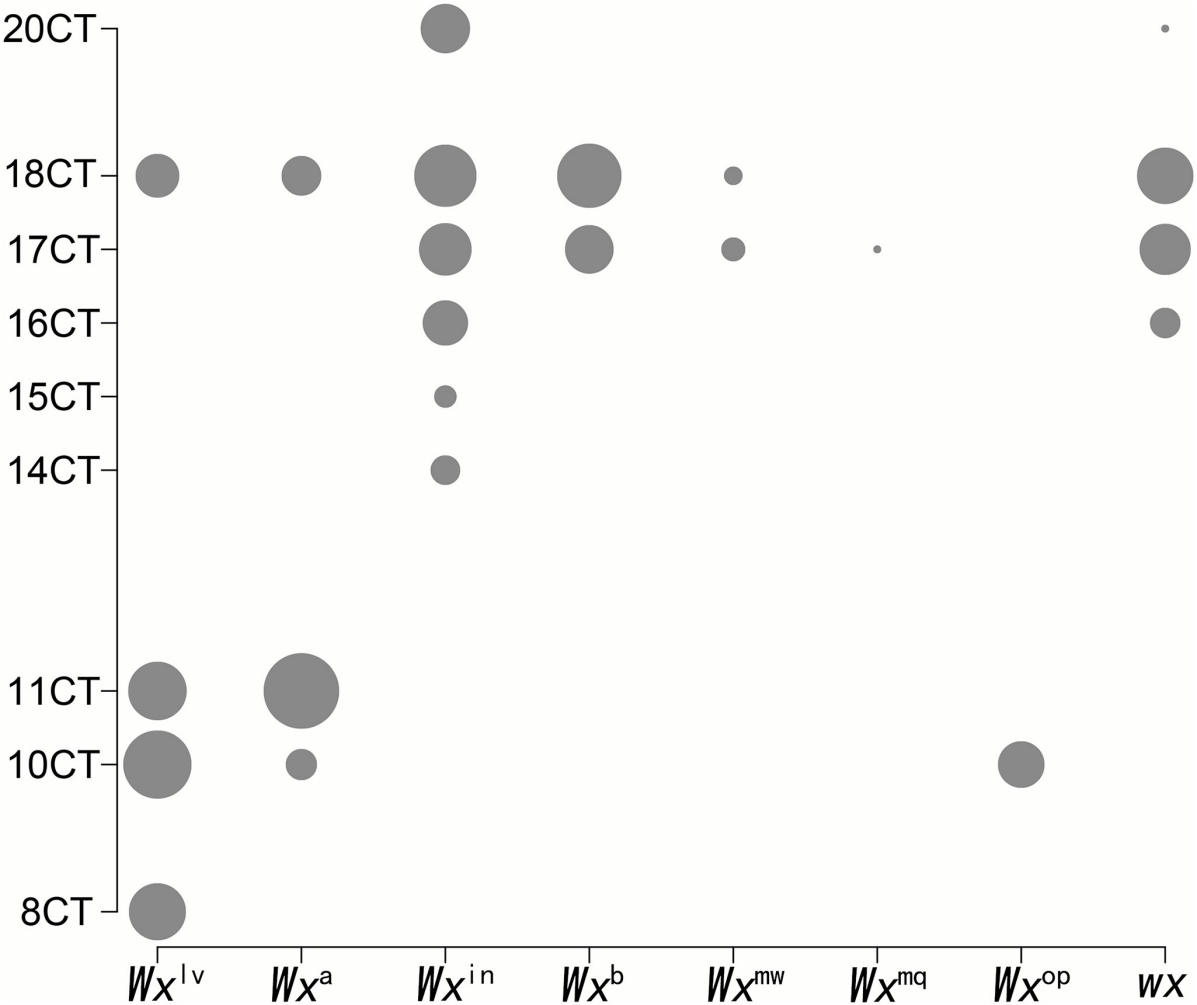
Relationship between the *Wx* alleles and number of CT repeats. The size of each grey dot represents the amount of each allele that corresponds to different CT repeats.

In most prior studies, AC was primarily defined according to the CT repeat. Generally, varieties with 17 or 18 CT repeats are considered to be “low-amylose” types, while those with 10 or 11 CT repeats are generally classified as “high-amylose” types [17]. Although previous studies found a significant correlation between the number of CT repeats and AC, some related molecular markers have been developed. Nonetheless, a considerable amount of AC variation could not be explained by the CT repeats. By analysing the number of CT repeats corresponding to different *Wx* alleles in the 3K data, we found that most low-amylose varieties had long CT repeats, while some high-amylose varieties had short CT repeats. These results are consistent with the findings of previous studies [17,29]. However, some high-amylose alleles, such as *Wx*^*a*^ and *Wx*^*lv*^, also contained long CT repeats, and some low-amylose alleles, such as *Wx*^*op*^, had only short CT repeats (Fig 6). These exceptions help to explain the AC variation, which could not be explained by microsatellite markers.

### KASP genotyping of the *Wx* alleles in hybrid parents

**Fig 7:**
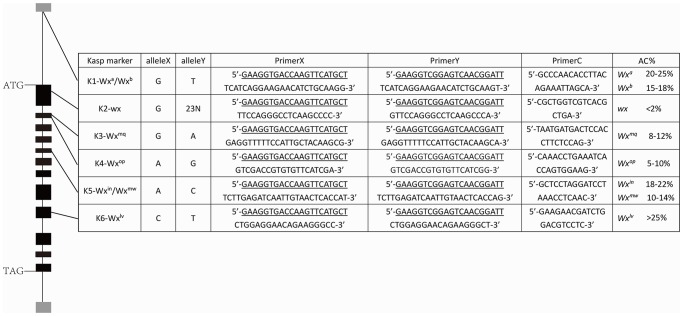
Primer sequences for KASP genotyping of different *Wx* alleles. Primers X and Y are two allele-specific primers (one for each SNP allele), and each primer contains 5ʹ fluoro-labelled oligos (underlined). Primer C represents a common (reverse) primer. Alleles X and Y represent SNPs or InDels at the 3ʹ end of primers X and Y, respectively. AC% represent amylose content of each allele.

**Fig 8:**
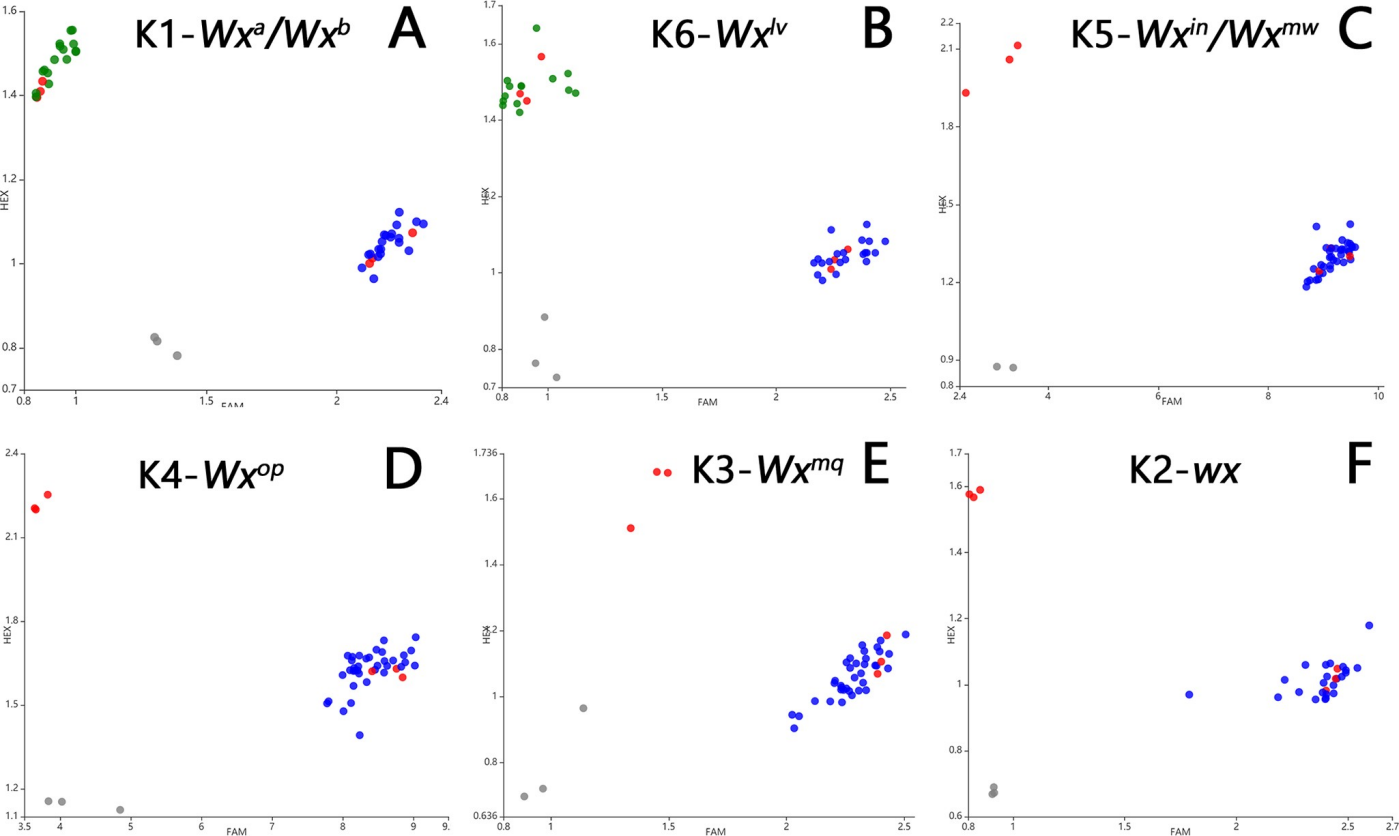
KASP genotyping of the different *Wx* alleles in main parent lines. Scatter plots of the selected KASP assays reveal the clustering of varieties on the X- (FAM) and Y- (HEX) axes. Varieties coloured blue represent the FAM-type allele, while those coloured green represent the HEX-type allele. Red dots represent the control groups, and black dots represent the non-template control (NTC). (**A**) KASP genotyping for the In1+1 SNP (G/T), Zhenshan 97A (*Wx*^*a*^) and Nipponbare (*Wx*^*b*^) were used as the control group. (B) KASP genotyping for the Ex10+115 SNP (C / T), Kasalath (*Wx*^*lv*^) and Zhenshan 97A (*Wx*^*a*^) were used as the control group. (C) KASP genotyping for the Ex6+62 SNP (A/C); Basmati (*Wx*^*in*^) and Nipponbare (*Wx*^*b*^) were used as the control group [13]. (D) KASP genotyping for the Ex4+77 SNP (A/G); Haopi (*Wx*^*op*^) and Nipponbare (*Wx*^*b*^) were used as the control group [9]. (E) KASP genotyping for the Ex4+53 SNP (G/A); Nanjing 46 (*Wx*^*mq*^) and Nipponbare (*Wx*^*b*^) were used as the control group [30]. (F) KASP genotyping for the 23-bp insertion at the Ex2+88 site; Suyunuo (*wx*) and Nipponbare (*Wx*^*b*^) were used as the control group [31].

**Fig 9:**
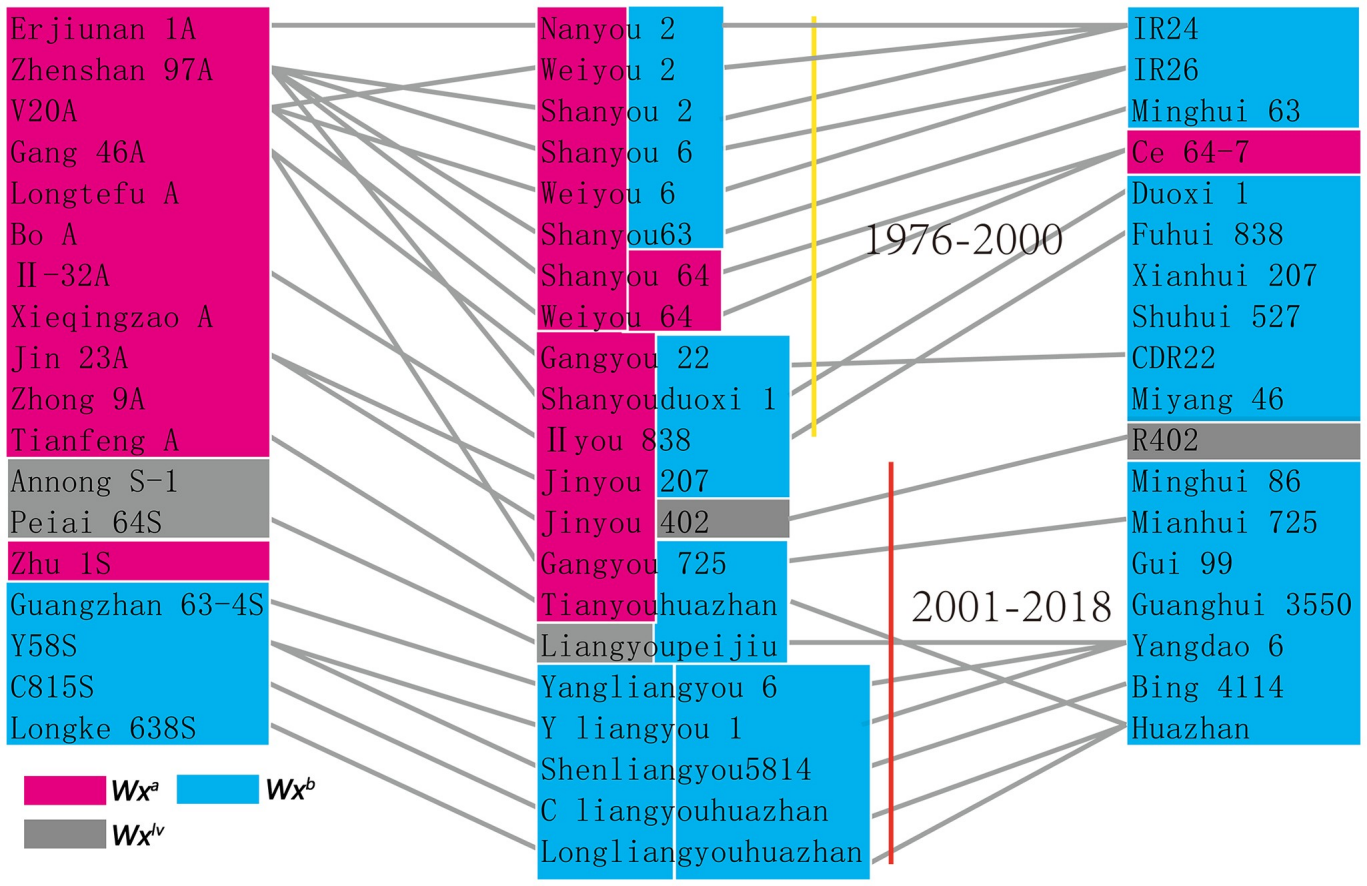
Genotyping of *Wx* in the hybrid parents. The left column represents the sterile line, right column represents restorer lines, and middle column represents hybrid combinations. Each vertical line with a unique colour represents different time periods in the development of hybrid rice.

Considering that these eight known *Wx* alleles covered 99.9% of the allelic variation, a set of KASP markers was developed based on these *Wx* alleles (Fig 7). Thirty-six main parent lines, including 18 female parents and 18 male parents of hybrid rice from 1976 to 2018 [20], were selected for genotyping (Fig 8). Fig 9 indicates that *Wx*^*b*^ is the major allele for nearly all restorer lines. *Wx*^*a*^ is the major allele involved in the male sterility of three-line hybrid rice, such as Zhenshan97A. *Wx*^*lv*^ is the major allele involved in the male sterility of early two-line hybrid rice, such as Annong S-1 and Peiai 64S, and with the development of hybrid rice, *Wx*^*lv*^ was gradually replaced by *Wx*^*b*^.

## Discussion

This study identified five new allelic variations for the first time. By determining the AC in four lines that contained these five variation sites, we found that only the AC of IRIS_313–10892 (Ex10+101) changed significantly compared with the AC of the wild type. Analysis of the crystal structure of OsGBSS1 showed that Lys413 and Arg408 are two important amino acids that directly interact with ADP, and Glu410 was found to be linked with these two amino acids via hydrogen bonds. Previously, many artificial point mutations in OsGBSS1 have been identified, includeding E410 [6]. In their study, Glu410Asp and Glu410Gln resulted in a reduction in AC and OsGBSS1-specific activity. Glu410Gly was a natural allelic variation, where the acidic amino acid was replaced by a non-polar amino acid without any R group; this type of change would probably have a strong effect on the conformation of Lys413 and Arg408. Except for *wx* in glutinous rice, *Wx*^*op*^ is currently the allelic variation with the lowest AC in non-glutinous rice. In this study, we found a novel allele with AC between *wx* and *Wx*^*op*^, which increased the diversity of the variation in AC in cultivated rice.

By analysis of the *Wx* allelic variation in thirty-six main parents of hybrid rice from 1976 to 2018, we found that only three *Wx* alleles existed, and the allelic combinations in hybrid rice changed from *Wx*^*a*^/*Wx*^*b*^ and *Wx*^*lv*^/*Wx*^*b*^ to *Wx*^*b*^/*Wx*^*b*^ as quality improved. *Wx*^*a*^ and *Wx*^*lv*^ are two alleles with high amylose content and hard texture of cooked rice, and *Wx*^*b*^ is a low-amylose allele with higher taste value [7]. Therefore, removing these two alleles and introducing *Wx*^*b*^ might be the main reason for the quality improvement of hybrid rice. In addition, With homozygosis of the *Wx*^*b*^ allele in hybrid rice, the AC of each grain in the hybrid combination tends to be more homogeneous. However, as high temperatures occur frequently in the southern regions of China, the AC of hybrids in these regions decreased significantly, which have strongly affected their appearance and eating quality. Many studies revealed that the splicing efficiency of the first intron of *Wx* is affected by temperature [32–36]. This might be the important reason why *Wx*^*b*^ is sensitive to high temperature. *Wx*^*in*^ is an intermediate amylose allele with the G base at the leader intron splice site. In addition, our analysis found that *Wx*^*in*^ is the predominant allele in the circum-Basmati group (cB), which includes many high-quality rice varieties, such as Basmati and Sadri aromatic varieties. Thus, we suggest that *Wx*^*in*^ is the preferred choice for improving the quality of *indica*-type rice in southern China.

As only three allelic variations were present in the hybrid rice, more favourable *Wx* alleles from cultivated rice should be identified, introduced, and effectively used to improve hybrid rice quality in the future. By evaluating the genetic effects of different allelic combinations, appropriate genotypic combination to achieve improved rice quality can be identified.

## Supporting information

S1 Appendix (XLS)

S2 Appendix (XLSX)

## Abbreviations

AC: amylose content
cA: circum-Aus
CAM: Central America and Caribbean
cB: circum-Basmati
CNCGB: China National Crop Genebank
CT: Cytosine and thymidine
dCAPS: derived cleaved amplified polymorphic sequence
EAF: East Africa
EAR: East Asia Islands
EAS: East Asia
EUR: Europe
GJ: *Geng*/Japonica
IOC: Indian Ocean
IRGC: International Rice Genebank Collection
KASP: Kompetitive allele-specific PCR
NAF: North Africa
NAM: North America
NTC: non-template control
OCE: Oceania
OsGBSS1: granule-bound starch synthase
RFGB: Rice Functional Genomics-based Breeding
SAC: South Asia–Central
SAE: South Asia–East
SAM: South America
SAW: South Asia–West
SEA: Southeast Asia
SER: SEA islands
WAF: West Africa
WAS: West Asia
XI: *Xian*/Indica
